# Cushing's syndrome as a paraneoplastic feature of renal cell carcinoma: A case report

**DOI:** 10.1016/j.eucr.2023.102504

**Published:** 2023-07-13

**Authors:** Asaad Moradi, Nasrollah Abian, Behnam Shakiba, Kiarash Moradi, Leyla Arefian

**Affiliations:** a Department of Urology, Firoozgar Hospital, School of Medicine, Iran University of Medical Sciences, Tehran, Iran; b Department of Urology, 5 Azar Hospital, School of Medicine, Golestan University of Medical Sciences and Health Services, Gorgan, Iran; c Department of Pathology, Aban General Hospital, Tehran, Iran

**Keywords:** Cushing's syndrome, RCC, ACTH, Paraneoplastic syndrome

## Abstract

Cushing's syndrome has been believed to be a paraneoplastic syndrome of renal cell carcinomas. However, there appears to be a dearth of compelling evidence to substantiate this notion. The only eligible documentation of Cushing's syndrome due to ectopic adrenocorticotropic hormone secretion by renal cancer in English literature dates back to 1988, and it pertains to a deceased patient discovered during an autopsy. Here, we present the first case of Cushing's syndrome as a paraneoplastic feature of renal cancer which showed complete resolution following surgical removal of the tumor. Additionally, we conduct review of the literature on this particular subject.

## Highlights

• Data supporting Cushing's syndrome as paraneoplastic syndrome of renal cancer are scarce.
• Renal cancer might cause Cushing's syndrome by producing ectopic ACTH.
• Paraneoplastic Cushing's syndrome may be relieved after partial nephrectomy as happened in our case report.

## 1 Introduction

Paraneoplastic syndromes are observed in 10%–20% of renal cell carcinomas (RCC). They can be broadly classified into endocrine and non-endocrine forms.^1^ Hypertension, hypercalcemia, anemia, and polycythemia are among the well-documented paraneoplastic syndromes associated with RCC. Cushing's syndrome has traditionally been regarded as a rare paraneoplastic syndrome in RCC patients; however, surprisingly, sufficient evidence supporting this claim has never been provided. Here, we present a case of Cushing's syndrome as a paraneoplastic feature of RCC and provide a literature review on this particular issue.

## 2 Case presentation

**Fig. 1:**
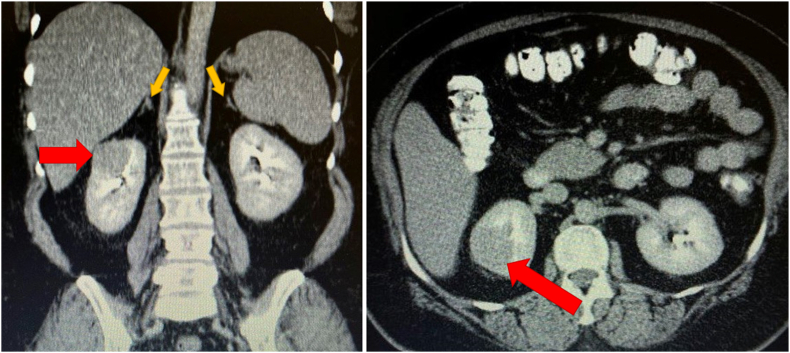
Abdominopelvic CT scan with intra-venous and oral contrast, showing right renal mass (red arrows) and normal adrenal glands (yellow arrows). (For interpretation of the references to colour in this figure legend, the reader is referred to the Web version of this article.)

Our patient was a 47-year-old woman with poorly controlled diabetes and hypertension. Her blood pressure was 150/90, her BMI was 36, and she had a moon-face appearance on physical examination. She was taking metformin and losartan for her diabetes and hypertension, respectively, and denied taking any form of exogenous steroids. Considering the possibility of Cushing's syndrome, a low-dose dexamethasone suppression test was performed, which revealed high serum cortisol levels. A 24-h urine sample indicated cortisol levels of 740 μg/24-h urine (normal: 45 μg/d), while metanephrine and vanillylmandelic acid levels were normal. Further assessments by the endocrinologist suggested that ectopic adrenocorticotropic hormone (ACTH) might be the cause of this syndrome, with the ACTH level measuring 84 pg/mL (normal: 10-60 pg/mL). As a result, she underwent a chest and abdominopelvic CT scan with and without IV contrast. Her lungs and adrenal glands appeared normal on CT scan, but a 4 cm enhancing mass on her right kidney was observed (Fig. 1). Our evaluations found no alternative source for ectopic ACTH. Following consultation with the endocrinologist and preoperative preparations, the patient underwent partial nephrectomy. Subsequently, she was admitted to the intensive care unit (ICU). Notably, there were no significant blood pressure fluctuations observed post-surgery. The pathology report indicated clear cell RCC with PT1a staging and clear surgical margins. Two months after the surgery, her blood pressure and blood sugar levels returned to the normal range, along with normalization of her morning serum cortisol, ACTH, and 24-h urine cortisol levels.

## 3 Discussion

While paraneoplastic syndromes are observed in up to 20% of RCCs, the occurrence of Cushing's syndrome as a paraneoplastic syndrome is rare. The first report of this association was published in 1961 by Riggs et al., who demonstrated the possibility of Cushing's syndrome due to RCC in 3 patients.^2^ However, Azzopardi et al. later questioned the validity of these cases, stating that none of the characteristic features of ‘malignant Cushing's syndrome’ were recorded and that there were no acceptable cases of renal carcinoma causing Cushing's syndrome.^3^ It was not until 1988, when Watanobe et al. reported the first well-documented case of Cushing's syndrome due to RCC in a deceased patient following autopsy.^4^ Although Cushing's syndrome has since been recognized as a paraneoplastic feature of RCC, no further evidence has been provided.

Ectopic sources of ACTH, originating from organs other than the pituitary gland, account for 10-15% of cases of ACTH-dependent Cushing's syndrome. Bronchial and lung cancers are the most common sources, but other organs can also be implicated.^1^ When investigating ectopic ACTH as a cause of Cushing's syndrome, cross-sectional imaging is typically the initial modality for localizing the source. CT scan has a sensitivity of 66–81% in this regard. Recently, a new ^68^Ga-DOTATATE PET/CT imaging technique has shown promising results in identifying ectopic sources of ACTH.^5^ However, this scan is not available in our country.

In our case, a middle-aged woman was diagnosed with RCC by CT scan after evaluation for ACTH-dependent Cushing's syndrome. No other source of ectopic ACTH, aside from the kidney tumor, was identified, as all other CT scan findings were normal. The preferred treatment for paraneoplastic syndromes in RCC is surgery, unless the patient has hypercalcemia. Therefore, considering the possibility of our patient's condition being a paraneoplastic syndrome, a partial nephrectomy was performed. After the surgery, the symptoms associated with Cushing's syndrome were alleviated and all laboratory test results in this regard returned to normal.

To our surprise, we found very limited evidence in the English literature regarding Cushing's syndrome as a paraneoplastic feature of RCC. While we found our case to be an interesting presentation of RCC, we did not anticipate having to thoroughly examine English data from over fifty years ago until the present time. To the best of our knowledge, this is the first reported case of Cushing's syndrome as a paraneoplastic feature of RCC in English literature, which demonstrated complete resolution following the surgical removal of the RCC.

## 4 Conclusion

While Cushing's syndrome has many etiologies, urologists must be aware that it might present itself as paraneoplastic syndrome of RCC, rendering the need for surgery as soon as possible. Also, any uncommon finding in practice can be a hint to researcher to review the literature as surprising results might ensue.

## Declaration of competing interest

None.
